# Evaluation of IL-17 and IL-35 Serum Levels in Patients with Preeclampsia

**Published:** 2019

**Authors:** Atefeh Batebi, Bahia Namavar-Jahromi, Mohammad Ali-Hassanzadeh, Moslem Ahmadi, Mahsa Sadat Hosseini, Behrouz Gharesi-Fard

**Affiliations:** 1- Department of Gynecology and Obstetrics, School of Medicine, Shiraz University of Medical Sciences, Shiraz, Iran; 2- Infertility Research Center, School of Medicine, Shiraz University of Medical Sciences, Shiraz, Iran; 3- Department of Immunology, School of Medicine, Shiraz University of Medical Sciences, Shiraz, Iran; 4- Department of Immunology, School of Medicine, Jiroft University of Medical Sciences, Jiroft, Iran

**Keywords:** Interleukin-17, Interleukin-35, Pre-eclampsia, Pregnancy, Proteinuria

## Abstract

**Background:**

Pre-eclampsia (PE) is the most common pregnancy complication affecting 2–8% of all pregnancies. PE could lead to maternal and prenatal morbidity. Imbalanced cytokine network and altered levels of several inflammatory and anti-inflammatory cytokines have been reported in PE. Because of scare information regarding the roles of IL-17 and IL-35 in PE, the current study aimed to investigate the serum level of these cytokines in a group of Iranian women suffering from PE.

**Methods:**

Serum samples were collected from 100 pre-eclamptic and 100 healthy pregnant women. Patients and controls were matched for age, ethnicity and body mass index. The level of IL-35 and IL-17 were evaluated by ELISA technique. T test and one-way ANOVA with Tukey Post-Hoc test were used for analysis and p<0.05 were assumed significant.

**Results:**

The serum level of IL-35 was increased in pre-eclamptic subjects as compared with healthy pregnant women (p<0.001). There was no significant difference in the serum level of IL-17 between pre-eclamptic and healthy pregnant women (p=0.73). Moreover, the results of the present study also showed that the pregnant women with severe pre-eclampsia had higher level of IL-35 in their sera when compared to those with mild form of the disease (p<0.001). In addition, the serum level of IL-35 was significantly elevated in women with higher proteinuria (p<0.001).

**Conclusion:**

Based on the our results, it seems that elevated levels of IL-35 in sera of pre-eclamptic women might work as a marker to evaluate the severity of the preeclampsia.

## Introduction

Pre-eclampsia (PE) is the most common pregnancy complication affecting about 2–8% of pregnancies. PE is the main cause of pregnancy related death in developed countries. In addition to morbidity and mortality in pregnant women, PE could lead to prenatal death, preterm birth and also intrauterine growth restriction (1). Clinically, PE is defined by gestational hypertension after 20 weeks of gestation along with proteinuria or along with significant end-organ dysfunction (2). Although the exact etiology for the disease is not well known but there is no doubt that PE is a disease of placenta. In women with PE, reduced trophoblast invasion and defective uterine spiral artery remodeling are detected (3). Immune system plays a major role in establishment of a normal pregnancy. The presence of immune cells in feto-maternal interface and their roles in a normal pregnancy have been clarified in details (4). In case of PE, several studies have shown modified frequencies of immune cells in pre-eclamptic women compared to those with a normal pregnancy. T lymphocytes and their derived cytokines play important role in providing a proper microenvironment for normal placentation and pregnancy. In line with the role of T cells in PE, increased frequencies of Th17, Th1 and decreased frequencies of Th2, Treg cells have been reported in pre-eclamptic women (5, 6). Cytokines are the main mediators for communication in the immune system. In the pregnancy period, cytokines act in a network manner and fine tuning of this network is crucial for a normal pregnancy. Imbalanced cytokine production has been reported in several pregnancy related disorders including PE (7). Dysregulation of inflammatory to anti-inflammatory cytokines in both feto-maternal tissues and maternal sera have been observed in preeclamptic patients (8). IL-17 and IL-35 are two cytokines with antagonist effects secreted by various immune or non-immune cells (9, 10). IL-17 family consists of 6 different cytokines (A to F) which are mainly produced by Th17 cells and generally play inflammatory roles. Among these cytokines, IL-17A is the founding member and the most studied one (11). In normal pregnancy, trophoblast cells express controlled level of IL-17 which seems to be critical for maintaining immune hemostasis in feto-maternal interface (12). In addition, elevated level of IL-17 in third trimester occurs along with normal term labor but uncontrolled deviation of Th17 and in turn uncontrolled expression of Il-17 is associated with several pregnancy complications such as preterm birth and PE (13, 14). Elevated proportion of Th17 cells in feto-maternal interface is considered as one of the main features of PE (15). Moreover, increased level of IL-17 in both sera and feto-maternal tissues of pre-eclamptic women has been reported (16).

IL-35 as a member of IL-12 family was first introduced as an anti-inflammatory cytokine mainly produced by Tregs (17). Recently, it has been shown that trophblast cells continuously produce IL-35 during normal gestation which is a critical cytokine to maintain feto-maternal tolerance (18). As mentioned, elevated expression of IL-35 during pregnancy is in favor of healthy and normal gestation while reduced level of its expression is conducive to pregnancy complications such as abortion (19). Worthy of note that two recently published investigations on preeclampsia reported reduced production of IL-35 in pre-eclamptic women (20, 21). Additionally, no studies have been launched to investigate the level of these cytokines in Iranian patients with pre-eclampsia. According to mentioned data, the current study aimed to investigate the serum level of IL-17 and IL-35 in a group of Iranian women with pre-eclampsia.

## Methods

### Subjects and sampling:

This study was conducted on 100 women diagnosed with PE as cases and 100 healthy pregnant women as the control group. Cases were selected among women who referred to Hafez and Zynabiyeh gynecology hospitals affiliated to Shiraz University of Medical Sciences, Shiraz, Iran. Inclusion criteria for cases were the presence of at least 1+ dipstick or 0.3 *gr* protein in 24-hour urine along with at least 140 mmHg systolic or 90 *mmHg* diastolic blood pressure. Moreover, based on the blood pressure and proteinuria, two groups of cases were selected. 54 women with at least 5 *gr* proteinuria and blood pressure above 160/110 *mmH*g were selected as severe PE group and 46 were labeled as mild PE group. Women with history of blood pressure before pregnancy, autoimmune disease, malignancy, and active infection were excluded from the study. Inclusion criteria for control group were the same as cases except for PE symptoms. It should be noted that all the patients had received medications. This study was approved by the local Ethics Committee of Shiraz University of Medical Sciences, Iran (IR.SUMS. REC.1395.S513). After signing the informed consents, 2 *ml* of peripheral blood were collected from all participants. Sera were separated and stored in aliquots at −70*°C* till performing the ELISA tests.

### Cytokine assay:

In order to evaluate the serum level of IL-17 and IL-35, enzyme-linked immunosorbent assay (ELISA) method was utilized. IL-17 was assessed using Human IL-17A (homodimer) ELISA Ready-SET-Go (eBioscience, USA, California.) according to manufacturer’s instructions and recommended concentrations. Briefly, 100 *μl* of serum samples were added to intended wells and incubated overnight at room temperature. After that plate was washed and then biotin-antibody was added and incubated for an hour at 37*^o^C*. Upon a triplicate washing, HRP-avidin was added to wells and incubated for another hour at 37*^o^C*. After that, wells were washed 5 times and substrate-TMB was added to the wells and incubated in dark at 37*^o^C* for 15 *min*. At final step, stop solution was added to wells and the optical density of each well was checked at 450 *nm* using ELISA reader. The sensitivity of the IL-17A kit was 1 *pg/ml*. In case of IL-35, human IL-35 ELISA kit (CUSABIO, USA, Texas) was used according to manufacturer’s instructions. The whole process was similar to IL-17A evaluation with one exception where after adding the serum samples, plates were incubated for 2 *hr* at 37*oC*. The sensitivity of IL-35 kit was 15.6 *pg/ml*.

### Statistical analysis:

SPSS statistical software, version 16 (SPSS Inc, Chicago, IL, USA) and oneway ANOVA with proper post hoc tests and student T test were used for data analysis and P-values less than 0.05 were considered statistically significant. Moreover, graphs were designed using ghraphPad PRISM software version 5 (GraphPad Software Inc, USA, San Diego).

## Results

Demographic data for all participants are presented in table 1. As shown, there were no statistical differences between cases and controls regarding age (30.02±5.88 *vs*. 29.11±5.56). Moreover, there were no significant differences between severe and mild preeclamptic women regarding age (29.65±6.1 *vs* 30.36±5.6). Based on the level of blood pressure and proteinuria, 54 and 46 cases were diagnosed as severe and mild preeclampsia, respectively. The level of blood pressure was significantly different between cases and controls as well as between severe and mild group (Table 1).

**Table 1.** Demographic and clinical characteristics of all studied groups

**Parameters**	**Healthy pregnant women**	**PE**	**p-value**	**Mild PE**	**Sever PE**	**p-value**
**Age (years)**	29.11±5.56	30.02±5.88	0.55	30.36±5.65	29.65±6.1	0.45
**SBP (*mmHg*)**	81.8±1.9	89.89±5.4	0.001	88.11±4.82	91.95±5.42	0.001
**DBP (*mmHg*)**	123±2.7	145±8.7	0.001	141.60±3.63	149.56±10.74	0.001

SBP= Systolic blood pressure, DBP= Diastolic blood pressure, PE= Pre-eclampsia

Evaluation of IL-35 concentration indicated that the mean level of IL-35 was significantly higher in pre-eclamptic women as compared with the control group (729±335 and 483.9±242, respectively, p<0.001 (Table2)). Moreover, the level of this cytokine was significantly higher in subjects with severe preeclampsia when compared to healthy controls (927±332 and 483.9±242, respectively, p<0.001, (Table 2)). However, the difference between mild preeclampsia and controls was not statistically different (558±228 and 483.9±242, respectively, p=0.02 (Table 2)). Interestingly, a significant difference between severe and mild preeclampsia was observed regarding IL-35 level. Women with severe preeclampsia expressed higher level of IL-35 as compared with those with the mild form (927±332 and 558±228, respectively, p<0.001 (Table 2)).

**Table 2.** IL-35 and IL-17 serum levels in pre-eclamptic patients (Severe and mild) as compared to control group

**Parameters**	**Healthy pregnant women** **Group 1(n=100)**	**PE** **Group 2(n=100)**	**Mild PE** **Group 3(n=46)**	**Severe PE** **Group 4(n=54)**
**IL-35**	483.9±242^*^	729±335^*^	558±228^*^	927±332^*^
**IL-17**	2.86±1.69	2.79±1.26	3±1.2	2.5±1.29

*: each group is different with others (p<0.05). One-way ANOVA with post-hoc Tukey tests were used for statistical analysis. PE: Preeclampsia, HC: Healthy control.

The mean serum levels of IL-17A in pregnant women with preeclampsia and healthy pregnant women were 2.79±1.26 and 2.86±1.69, respectively. There was no significant difference in the level of IL-17A among women with mild (3±1.2) or severe preeclampsia (2.5±1.29) when compared with control group (p=0.14, p=0.32, respectively (Table 2)). The results also showed that there was no significant difference in the serum level of IL-17A between women with mild and severe preeclampsia (3±1.2 and 2.5±1.29, respectively, p=0.46 (Table 2)).

**Figure 1.**
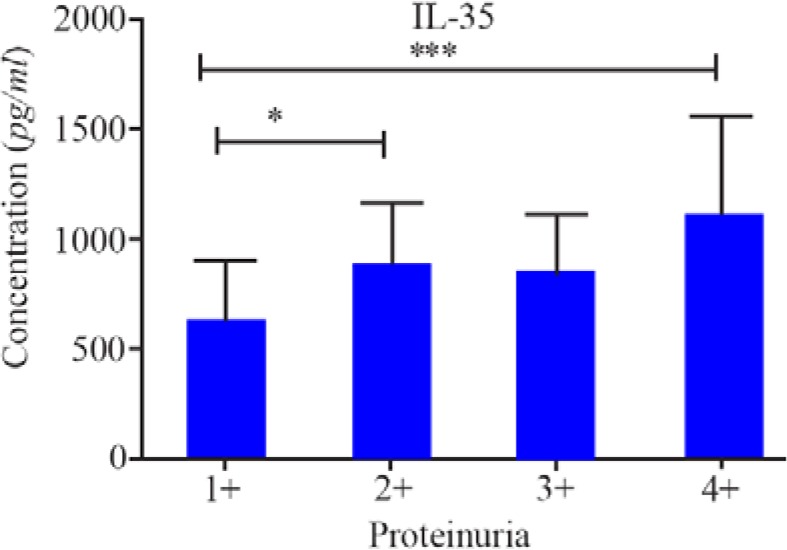
Comparison of IL-35 serum levels in pre-eclamptic patients with different proteinuria levels. * and *** represent p<0.05 and p<0.001, respectively.

In the next step, the relationship between the levels of IL-35 and IL-17A with blood pressure and proteinuria was compared in patients. Results indicated that the mean level of IL-35 was significantly higher in patients with 4+ proteinuria as compared to those with less than 4+ proteinuria (p<0.001) (Figure 1). There were no significant differences between the levels of IL-17A with blood pressure or proteinuria. Finally, no correlation was found between IL-35 and IL-17A levels in both cases and controls (data not shown).

## Discussion

In the current study, the serum levels of an anti-inflammatory cytokine (IL-35) and a Th17 related inflammatory cytokine (IL-17) were evaluated in a group of Iranian women with pre-eclampsia. The results of the present study indicated that the level of IL-35 was significantly increased within sera samples from pre-eclamptic women and also the increase was related to the severity of the disease. In addition, it was observed that pre-eclamptic women with higher level of proteinuria had elevated level of IL-35. It is well known that PE is a disease of placentation. Indeed, it is believed that a poor placentation in the beginning of pregnancy will lead to PE after the 20th week of gestation (22). To support a normal gestation from implantation to parturition, immune system works to balance inflammatory to anti-inflammatory responses in different time points of the pregnancy period. In the first trimester, especially at the early days of gestation, a controlled inflammatory response is needed to support implantation and placentation. At the second trimester, regulatory responses and later in the third trimester and finally at the end of the pregnancy again, inflammatory responses will dominate to support delivery. Moreover, cytokines do their function both in local and systemic manner (23). So, to investigate the immune responses and cytokine balances in pregnancy, time point and sampling are two important factors that might alter the obtained results. It is well known that pre-eclamptic women are in higher inflammatory state as compared with women with normal pregnancy. Higher expression of IL-35 during the last weeks of pregnancy, as seen in the present study in patients, may work as compensatory mechanisms to control the inflammatory responses. This finding could be explained by the results of IL-35 level as it is documented that IL-35 could suppress Th17 cells and inhibit IL-17 production (24). In line with this scenario, the level of IL-17 as an inflammatory cytokine in both cases and controls was the same. While only a few studies have been published regarding the expression of IL-17 and IL-35 in PE, there are controversies in the reported data. Ozkan et al. reported a decreased level of both IL-17 and IL-35 cytokines and also IL-35/IL-17 in women with PE (21). Moreover, Cao et al. studied the expression of IL-17 and IL-35 at both mRNA level (PBMC) and protein level (serum) in Chinese preeclamptic women (20). They also reported down-regulation in expression of IL-35 and up-regulation for IL-17 at both mRNA and protein levels. Interestingly, both mentioned studies have reported unbelievable low level of IL-35 within sera. Looking for the reported IL-35 level in normal and pregnancy related disorders indicates that the mean level of this cytokine is above 120 *pg/ml* while the level of this cytokine is reported in a range of 6.65–17 *pg/ml* by Ozkan and Coa et al. (19). Regarding the reported levels for IL-17 in both mentioned studies, the lower level of IL-17 can be seen as compared with other published papers (14). In case of IL-17, another point to be mentioned is the limitation of ELISA kit used by Ozkan et al. While they used an ELISA kit with assay range of 31–2000 *pg/ml*, they reported a range between 1.8–3.57 *pg/ml* for IL-17. Recently, our group have also reported declined level of IL-35 expression but in the placental tissues from pre-eclamptic women (25). In line with Cao et al.’s report, an elevated level of IL-17 was reported before, but only in blood samples collected from placenta (26). Interestingly, a recent study has reported elevated expression of EBI-3 (a chain of IL-35 heterodimer) in decidua from pregnant women with preeclampsia. They also showed increased level of HLA-G in pre-eclamptic women which is an anti-inflammatory agent. This group concluded that these increases may contribute in PE pathophysiology or may be the consequence of the disease (27). Moreover, increased level of IL-35 has been reported in several well-known diseases with inflammatory basis including diabetes, and inflammatory bowel disease (IBD) (28). The latest study has interpreted this increase as a compensatory mechanism of immune system to attenuate the effects of inflammation (29). The controversy is not limited to IL-35. For example, there are opposite reports regarding increasing or decreasing levels of IL-10 and IL-4 in PE (30–33). These controversial data necessitates the needs for more detailed investigations on cytokine network in PE. Our data also showed an association between the level of proteinuria and elevated level of IL-35. As far as searched, there was no study investigating the effects of IL-35 on proteinuria but in accordance with our findings, a published study on SLE patients reported an association between the levels of IL-35 with renal failure. However, it seems that more investigations are needed to find out if IL-35 is involved in renal problems directly or not (34). As mentioned, the results of the present study showed no difference between women with PE and healthy ones regarding IL-17 levels. This finding could be explained by the results of IL-35 levels as it is documented that IL-35 could suppress Th17 cells and inhibit IL-17 production (24). If the elevated level of IL-35 in these patients as an anti-inflammatory and compensatory reaction of immune system is observed, so the invariable level of IL-17 could also be attributed to this compensatory mechanism. There are some points that should be mentioned here to help for a better understanding of the current study and also other studies on PE. The first point that should be mentioned is that all patients which were enrolled in this study were under supervision of physicians and many of them received medications to control their disease. Moreover, it should be considered that the serum level of these cytokines was tested while the main scenario of pre-eclampsia pathology might take place in feto-maternal interface. Last but not least, it should be noted that immune system behavior in pregnancy is completely time-dependent and this finding could just be attributed to last days of pregnancy as our patients were referred to hospital for labor and delivery. Myatt et al. have published a paper on criteria and strategies for designing a perfect study on PE and mentioned these points and others such as age, previous disease and smoking that may influence the resultant data (35). Investigating the serum levels of the mentioned cytokines along with other cytokines during all periods of pregnancy will certainly provide more detailed and precise data on relation of these cytokines to the disease pathophysiology.

## Conclusion

In all, although high level of IL-35 may be associated with pre-eclampsia and severity of the disease but due to lots of unanswered problems, it is not suitable for evaluating the onset or severity of PE. Also, in case of its role in pathology of PE, there are lots of questions remained to be answered.
